# HER2 and GATA4 are new prognostic factors for early-stage ovarian granulosa cell tumor—a long-term follow-up study

**DOI:** 10.1002/cam4.230

**Published:** 2014-03-29

**Authors:** Anniina Färkkilä, Noora Andersson, Ralf Bützow, Arto Leminen, Markku Heikinheimo, Mikko Anttonen, Leila Unkila-Kallio

**Affiliations:** 1 Department of Obstetrics and Gynecology, University of Helsinki and Helsinki University Central HospitalHelsinki, Finland; 2 Children's Hospital, University of Helsinki and Helsinki University Central HospitalHelsinki, Finland; 3 Department of Pathology, University of Helsinki and HUSlab, Helsinki University Central HospitalHelsinki, Finland; 4 Department of Pediatrics, Washington University School of Medicine, St Louis Children's HospitalSt Louis, Missouri

**Keywords:** GATA4, granulosa cell tumor, HER2, prognostic factor, recurrence, survival

## Abstract

Granulosa cell tumors (GCTs) carry a risk of recurrence also at an early stage, but reliable prognostic factors are lacking. We assessed clinicopathological prognostic factors and the prognostic roles of the human epidermal growth factor receptors (HER 2–4) and the transcription factor GATA4 in GCTs. We conducted a long-term follow-up study of 80 GCT patients with a mean follow-up time of 16.8 years. A tumor-tissue microarray was immunohistochemically stained for HER2–4 and GATA4. Expression of HER2–4 mRNA was studied by means of real time polymerase chain reaction and *HER2* gene amplification was analyzed by means of silver in situ hybridization. The results were correlated to clinical data on recurrences and survival. We found that GCTs have an indolent prognosis, with 5-year disease-specific survival (DSS) being 97.5%. Tumor recurrence was detected in 24% of the patients at a median of 7.0 years (range 2.6–18 years) after diagnosis. Tumor stage was not prognostic of disease-free survival (DFS). Of the molecular prognostic factors, high-level expression of HER2, and GATA4, and high nuclear atypia were prognostic of shorter DFS. In multivariate analyses, high-level coexpression of HER2 and GATA4 independently predicted DFS (hazard ratio [HR] 8.75, 95% CI 2.20–39.48, *P* = 0.002). High-level expression of GATA4 also predicted shorter DSS (HR 3.96, 95% CI 1.45–12.57, *P* = 0.006). In multivariate analyses, however, tumor stage (II–III) and nuclear atypia were independent prognostic factors of DSS. In conclusion HER2 and GATA4 are new molecular prognostic markers of GCT recurrence, which could be utilized to optimize the management and follow-up of patients with early-stage GCTs.

## Introduction

Granulosa cell tumor (GCT) is a rare subtype of ovarian cancer, representing 3–5% of all ovarian malignancies. GCT presents with the juvenile and the more common adult subtype with mean age at diagnosis between 50 and 54 years 1. Adult GCTs are characterized by an indolent, albeit unpredictable, course of disease with a recurrence risk of 20–30% even with early-stage disease 2,3. Recently, adult GCTs were shown to have a distinct molecular background since a single somatic mutation (402C->G) in the gene encoding for Forkhead Box L2 (FOXL2) was found in 90–97% of GCTs 4–7. The functional role of the FOXL2 mutation in GCTs remains unresolved. Tumor stage (FIGO stages II–IV) is the only definitive prognostic factor affecting GCT recurrence and survival 2,8–10. However, in view of the fact that the majority of GCTs (80–90%) are diagnosed at stage I 3,8, new prognostic factors are needed.

Human epidermal growth factor receptors (HERs) are a group of transmembrane tyrosine kinase receptors including HER1, HER2, HER3, and HER4, which play essential roles in ovarian granulosa cell proliferation and survival 11,12. Overexpression of HER2 (ErbB2/neu) is prognostic of more aggressive disease in several cancers, including breast, gastric, and epithelial ovarian carcinomas 13–15. Moreover, HER2 can be therapeutically targeted with a monoclonal antibody trastuzumab and a small molecule HER2/EGFR (epidermal growth factor receptor) inhibitor lapatinib in the treatment of HER2 overexpressing breast and gastric cancers 16. Previously, few studies have assessed the expression of HER2, HER3, and HER4 in GCTs, and found GCTs positive for HER3 and HER4 17–20. The expression of HER2 in GCTs remains controversial; three studies revealed positive expression of HER2 in GCTs 17,18,20, while five others did not 19,21–24. The prognostic significances of HER2, HER3, and HER4 expressions and *HER2* gene amplification in GCTs are unknown.

GATA4 is a zinc-finger transcription factor that plays a crucial role in ovarian and granulosa cell development and function 25,26. The majority of GCTs express GATA4, and high-level expression is associated with higher tumor stage and increased recurrence risk 27. Further, GATA4 has been suggested to have a role in GCT pathogenesis by inhibiting apoptosis through activating anti-apoptotic B-cell lymphoma-2 28,29.

The relative rarity and long natural history of GCTs has hindered the identification of solid prognostic factors to guide therapeutic decisions in early-stage disease. We have previously screened a tumor tissue microarray (TTMA) for multiple factors of possible prognostic significance 27–32. In search of new prognostic tools, we herein performed a long-term follow-up study to evaluate potential prognostic factors of GCTs. In addition to evaluating GATA4 as a potential prognostic factor, we characterized the expression levels of HER2-4, as well as gene copy numbers of *HER2* in GCTs and correlated the expression levels to tumor recurrence and survival in a cohort of 80 GCT patients.

## Material and Methods

### Patients and tumor samples

The Ethics Committees of Helsinki University Central Hospital (HUCH) and the National Supervisory Authority of Welfare and Health in Finland approved this study. The clinical data and tumor samples were collected from 80 primary GCT patients diagnosed at HUCH between 1971 and 2003; the median year of diagnosis was 1990. In the follow-up study, we invited all living patients no longer in controls to a clinical visit, and 31 of them were examined by way of gynecologic examination, Pap smear, pelvic ultrasonography and assay of serum markers. Informed consent was obtained from these patients in the follow-up study. Follow-up data on 20 living and 29 deceased patients were taken from hospital files and reliable follow-up data was available from all 80 patients. Follow-up was performed until death (*n* = 29) or May 2012; the mean follow-up time was 16.8 years (SD 9.1 years). The causes of death were collected from death certificates retrieved from the Finnish causes of death registry.

Tumor tissue samples from 26 primary and five recurrent GCT patients were collected for Real time polymerase chain reaction (PCR) analyses during 1990–2009. RNA was isolated from frozen tumor tissue as described previously 31. The RNA quality was assessed according to the instructions provided with an Agilent 2100 bioanalyzer Eukaryote Total RNA Nano kit (Agilent technologies, Santa Clara, CA).

### Immunohistochemistry and silver in situ hybridization

A previously constructed TTMA of 80 primary and 13 recurrent GCTs was utilized 27; all diagnoses were reevaluated as adult GCTs by an expert pathologist (R. B.). The 13 recurrent GCTs in the TTMA were analyzed only for expression of the factors and excluded from the recurrence and survival analyses. Tumor subtype and the degree of nuclear atypia were evaluated, and mitotic index was graded as “high” when there were ≥5 mitotic figures per 10 high-power fields (HPFs) and “low” when there were <5 mitotic figures per 10 HPFs 27. Paraffin-embedded sections of the TTMA were stained for expression of HER2 (sc-33684; Santa Cruz Biotechnology Inc., Dallas, TX) and phosphorylated (P-) HER2 (ab47755; Abcam, Cambridge, UK), HER3 (sc-415; Santa Cruz Biotechnology Inc.) and P-HER3 (#4791; Cell Signaling Technology Inc., Danvers, MA) and HER4 (sc-283; Santa Cruz Biotechnology Inc.) and P-HER4 (sc-33040, Santa Cruz Biotechnology Inc.) as described previously 27. Each sample was analyzed for the intensity of staining (all antigens) and the percentage of positive cells (GATA4), decided as a consensus of opinion of two researchers (N. A., M. A.). The tumors were grouped into high-level (>80% of positive cells showing high-level or intermediate staining intensity) or low-level expression groups, the latter also containing the nonstaining tumors. Silver in situ hybridization (SISH) of the TTMA for *HER2* gene copy number was conducted as described 33, with minor modification; hybridization was performed at 52°C for 10 h. The *HER2* copy numbers were evaluated as a consensus of opinion of three researchers (A. F., N. A., R. B.). Low-level amplification was defined as three to six *HER2* signals per nucleus.

### Real time PCR

RNA was reverse transcribed using a TaqMan reverse Transcription kit (Applied Biosystems, Branchburg, NJ) in 40-*μ*L reaction volume. The following primers were used for real time PCR: HER2, forward 5′-GCTGGCTCTCACACTGATA, reverse 5′-AGACAGTGCGCGTCAG; HER3 forward 5′-GCTCCCTTCACCCTCT, reverse 5′-T CCCAGGACACACTGC; and HER4 forward 5′-GGAAGGATCTGCATAGAGTCT, reverse 5′-TCCATGGCATGTGAGGA. The *β*-actin gene was used as a reference gene and the primers used were forward 5′-CTGACGGCCAGGTCATCAC and reverse 5′-CAGACAGCACTGTGTTGGC. SYBR Green PCR Master mix (Applied Biosystems) was utilized in a 20-*μ*L reaction volume. The annealing temperature was 60°C, with 40 cycles, plus the dissociation step. A standard curve method was applied and analyses were performed in triplicate using an ABI PRISM 7700 sequence detection system (Applied Biosystems) according to the manual.

### Data analysis

Disease-free survival (DFS) was determined as the time from primary tumor operation to the first recurrence. Disease-specific survival (DSS) was determined as the time from diagnosis to death from GCT; other causes of death were censored. Associations between the immunohistochemical data and clinical characteristics were analyzed by using contingency tabling and χ^2^ or Fisher's exact tests, as appropriate. Distribution of continuous variables were evaluated with Shapiro–Wilk's test and normally distributed variables were correlated with one-way analysis of variance (ANOVA) and Student's *t*-test (age at diagnosis), and non-normally distributed with Mann–Whitney test (follow-up time, RNA expression data). Univariate Cox regression analysis and Kaplan–Meier log-rank test were performed for DFS and DSS according to the methodology. The Kaplan–Meier plots are displayed leaving a minimum of five subjects at risk, except in Figure 2A. Multivariate analyses were conducted using nominal logistic regression for associations with recurrence and the Cox regression model was used for DFS and DSS. Significant factors in univariate analyses were included into the multivariate models. The analyses were performed with JMP 9.0.0 (SAS Institute Inc., Cary, NC) software. A *P-*value <0.05 (two-sided test) was considered significant.

## Results

### Adult GCT recurs even in the early stages

Patient characteristics and recurrences in 80 cases of primary GCTs are summarized in Table 1. Eighteen (24%) patients had a recurrence during the follow-up period; 15 (83%) of these were of stage I. The median time to recurrence was 7.0 years (range 2.6–18 years, mean 7.7, SD 4.5). Six (33%) of the recurrences occurred within 5 years, 14 (78%) within 10 years, and 17 (94%) within 15 years after the diagnosis. One patient had a recurrence after 18.4 years after the primary diagnosis. Tumor recurrence was associated only with a high degree of nuclear atypia; recurrence was not associated with any other factors in Table 1 (Fisher's exact test, data not shown). Follow-up times among the patients with recurrence were similar to those among those with no recurrence (*P* = 0.7).

**Table 1:** Clinical and tumor characteristics of 80 primary GCT patients

	Total (*n* = 80)	No recurrence (*n* = 62)	Recurrence (*n* = 18)
		
	*n* (% of total)	*n* (% of characteristic)	
(A) Patient characteristics			
Mp status			
PreMp	37 (46.2)	25 (67.6)	12 (32.4)
PostMp	43 (53.8)	37 (86.0)	6 (14.0)
Stage			
I	71 (88.8)	56 (78.9)	15 (21.1)
Ia	50 (70.4)	40 (80.0)	10 (20.0)
Ib	1 (1.4)	1 (100)	0 (0)
Ic	20 (28.2)	15 (75.0)	5 (25.0)
IO rupture*	12 (60.0)	8 (66.7)	4 (33.3)
II	6 (7.5)	4 (66.7)	2 (33.3)
III	3 (3.8)	2 (66.7)	1 (33.3)
Treatment			
Surgery only	65 (81.3)	50 (76.9)	15 (23.1)
Surgery + C	12 (15.0)	10 (83.3)	2 (16.7)
Surgery + R	1 (1.3)	0 (0)	1 (100)
Surgery + C + R	2 (2.5)	2 (100)	0 (0)
Age at diagnosis (years), median (range)	52 (19–87)	52 (19–87)	50 (28–76)
Follow-up time (years), median (range)	13.9 (0.1–36.7)	13.6 (0.1–36.7)	15.6 (4.5–34.0)
(B) Tumor characteristics			
Tumor size			
≥10 cm	32 (40.0)	23 (71.9)	9 (28.1)
<10 cm	48 (60.0)	39 (81.3)	9 (18.7)
Subtype			
Sarcomatoid	24 (30.0)	16 (66.7)	8 (33.3)
Differentiated	56 (70.0)	46 (82.1)	10 (17.9)
Nuclear atypia			
High	18 (22.5)	11 (61.1)	7 (38.9)
Low	62 (77.5)	51 (82.3)	11 (17.7)
Mitotic index			
High	20 (25.0)	13 (65.0)	7 (35.0)
Low	60 (75.0)	49 (81.7)	11 (18.3)

Mp, menopause; IO, intraoperative; C, chemotherapy; R, radiation therapy.

* IO tumor rupture as percentage of stage Ic tumors.

Survival data were available for all 80 patients. By the end of the follow-up period, 51 (63.8%) patients were alive, 11 (13.7%) had died of GCT, and 18 (22.5%) had died of other causes. The 5-, 10-, and 15-year DSS rates were 97.5%, 91.9%, and 89.9%, respectively. In stage I patients, 10-year DSS was significantly greater (95.8%, *P* = 0.02), than in stage II (83.3%) or stage III (33.3%) patients. The median time between diagnosis and death from GCT was 9.7 years (range 0.1–33.9 years). One patient with inoperable stage IIIc GCT died of the disease 3 weeks after primary tumor operation. If the tumor recurred, the 10-year DSS was 72.2%. The median time between the first recurrence and death was 6.4 years (range 0.3–20.4 years). Of the clinicopathological factors, only nuclear atypia was associated with DSS (*P* = 0.01); no other factors in Table 1 correlated to DSS (Fisher's exact test, data not shown).

### HER2, HER3, and HER4 are expressed in GCTs

**Figure 1:**
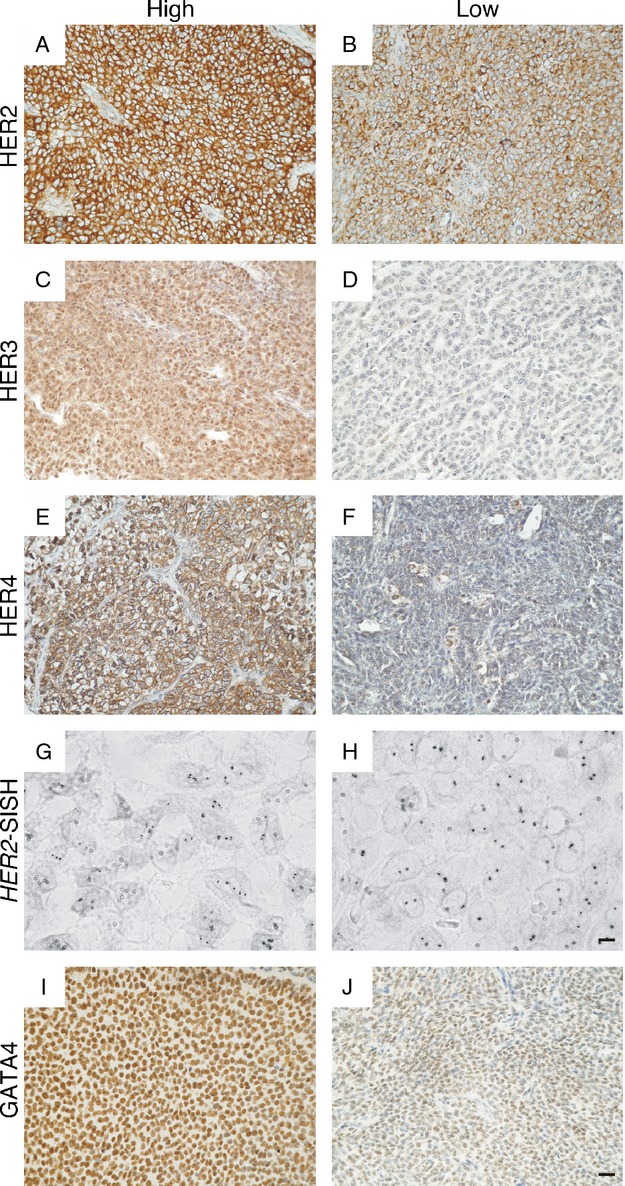
Representative images of HER2, HER3, HER4, and GATA4 immunohistochemical staining and *HER2* silver in situ hybridization in adult GCTs. A GCT sample with (A) high-level expression and (B) low-level expression of HER2 on the cell membrane. High-level expression of HER3 localized into the nucleus and cytoplasm (C). In the majority of GCTs HER3 immunostaining was negligible (D). HER4 was localized into the cell membrane in both (E) high-level and (F) low-level expressing GCTs. Eight GCTs showed small-scale amplification of the *HER2* gene (G), while gene copy number was normal in most tumors (H). High-level nuclear expression of the transcription factor GATA4 (I) and a GCT with low-level GATA4 expression (J). The magnification of (A–F, I, and J) is 20× and the scale bar represents 50 *μ*m. The magnification of (G and H) is 100× and the scale bar represents 10 *μ*m.

In a search for new prognostic tools, the TTMA was immunohistochemically stained for native and phosphorylated (P-) HER2-4; data for the native proteins are summarized in Table 2. We found positive staining for HER2 in 84 (98%) tumors and in 79 (90%) of the tumors, HER2 was also expressed in its phosphorylated form. P-HER3 was expressed in 8 (9%) of the tumors and P-HER4 in 74 (84%), respectively. We also analyzed gene copy number alterations of *HER2* by means of SISH in 91 GCTs and found low-level gene amplification (three to six signals) of *HER2* in eight primary GCTs. Low-level gene amplification was not associated with tumor size, stage, nuclear atypia, or recurrence. Data on GATA4 staining in 80 primary and 13 recurrent tumors have been presented previously 27. Based on the immunostaining, the tumors were divided into groups of high and low expression; representative images of the groups are presented in Figure 1. The expression levels of HER2–4 did not correlate to each other and there were no differences between the expression patterns of primary and recurrent GCTs. We found a strong positive correlation between HER2 and GATA4 protein levels in primary GCTs (*P* = 0.002), and also in all the tumors (*P* = 0.0006) (data not shown). In primary GCTs, high-level expression of both HER2 and high GATA4 correlated positively to higher tumor stage (stages II–III and Ib–III, *P* < 0.05). Furthermore, we found that high-level expression of HER2 (*P* = 0.02) and GATA4 (*P* = 0.006) were associated with tumor recurrence.

**Table 2:** Protein expression levels of 80 primary and 13 recurrent GCTs

Immunohistochemistry and SISH *n* (%)			
Factor	Expression level	Primary (*n* = 80)	Recurrent (*n* = 13)
HER2	High	17 (22.7)	4 (33.3)
	Low	58 (77.3)	8 (66.7)
HER3	High	18 (23.1)	8 (66.7)
	Low	60 (76.9)	4 (33.3)
HER4	High	54 (73.0)	10 (83.3)
	Low	20 (27.0)	2 (16.7)
*HER2* SISH	3–6 copies	8 (10.1)	0 (0)
	No amplification	71 (89.9)	12 (100)
GATA4	High	34 (42.5)	5 (38.5)
	Low	46 (57.5)	8 (61.5)
HER2 + GATA4	High H + high H	13 (18.0)	3 (25.0)
	High H + low G	1 (1.4)	1 (8.3)
	Low H + high G	20 (27.8)	2 (16.8)
	Low H + low G	38 (52.8)	6 (50.0)

SISH, silver in situ hybridization; H, HER2; G, GATA4.

In order to verify the mRNA expression levels of HER2–4 in GCTs, we performed real time PCR of HER2–4 in 31 GCTs. We found that both primary and recurrent tumors similarly expressed mRNA for HER2–4. The expression of HER2 was robust (median 1.1 ng, range 0.5–2.5 ng), whereas the levels of HER3–4 were low (HER3; median 0.3 ng, range 0.06–7.0, HER4; median 0.3 ng, range 0.03–7.0 ng). The RNA expression levels did not correlate to clinical parameters (e.g., primary tumor size, stage) or probability of recurrence (data not shown).

### HER2, GATA4, and nuclear atypia are prognostic of GCT recurrence

**Figure 2:**
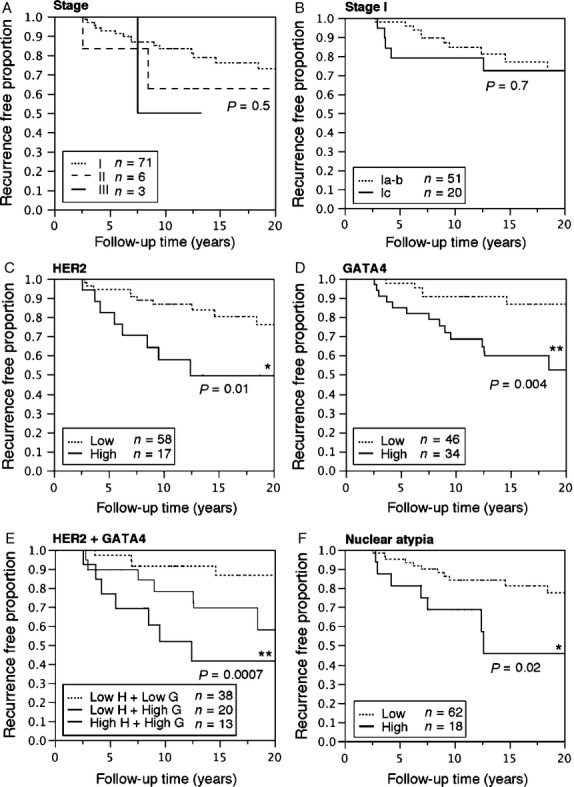
Kaplan–Meier plots of disease-free survival (DFS) to the first recurrence in 80 GCT patients according to tumor stage, expression of HER2 and GATA4, and nuclear atypia. Recurrence-free proportion of patients with either stage I, II, or III GCTs (A), and in patients with stage I tumors (B). The recurrence-free proportion of patients with tumors expressing low or high levels of HER2 (C), GATA4 (D), and in combinations (E) and in tumors having low-level or high-level nuclear atypia (F). H, HER2; G, GATA4. The Kaplan–Meier plot for the low G + high H group is not shown in E due to small sample size (*n* = 1), and the *P*-value in E is derived from analysis between the low H + low G versus the high H + high G expression groups. Log-rank test, differences between groups were considered significant when *P* < 0.05 (*P < 0.05, **P < 0.01).

The effects of stage, HER2, GATA4, and nuclear atypia on GCT prognosis were investigated by means of Kaplan–Meier analyses of DFS (Fig. 2). In our analyses, tumor stage was not prognostic as regards DFS (Fig. 2A and B). Within the first 5 years, however, there was an increased risk of recurrence among stage Ic patients compared with stage Ia–b patients (hazard ratio [HR] 10.64, 95% confidence interval [95% CI] 1.57–208.12, *P* = 0.01) (Fig. 1B). However, the difference was not significant after 10 or 15 years of follow-up.

High-level expression of HER2 (Fig. 2C, Table 3A) and GATA4 (Fig. 2D, Table 3A) were prognostic of shorter DFS, and DFS was even shorter if the primary tumor coexpressed high levels of both HER2 and GATA4 (Fig. 2E, Table 3A). Within 10 years, 48.1% (SE 14.3%) of the tumors coexpressing high-level GATA4 and HER2 had recurred, while only 8.7% (SE 4.7%) of the low-GATA4- and low-HER2-expressing tumors had recurred. Both high-level GATA4 and high-level HER2 predicted DFS independently of tumor stage (Table 3A). High-level nuclear atypia was also prognostic of shorter DFS (Fig. 2F), as it also was after adjusting for tumor stage (Table 3A).

**Table 3:** Univariate and multivariate Cox regression analyses regarding disease-free survival (DFS) and disease-specific survival (DSS) in 80 GCT patients

(A) Univariate and stage-adjusted risk factors for DFS					
Factor	Level	HR (95% CI)	*P*-value	AHR (95% CI)	*P*-value
HER2	High	3.15 (1.20–8.00)	0.01*	3.02 (1.11–7.94)	0.03*
GATA4	High	4.04 (1.52–12.64)	0.004**	3.96 (1.45–12.57)	0.006**
HER2 and GATA4	High + high	6.61 (1.98–25.44)	0.002**	6.40 (1.78–25.6)	0.005**
Nuclear atypia	High	3.00 (1.10–7.66)	0.02*	3.21 (1.17–8.03)	0.03*

(B) Multivariate; Independent risk factors for DFS					
Factor	Level	HR (95% CI)	*P*-value	AHR (95% CI)	*P*-value
HER2	High	2.19 (0.79–6.00)	0.1	2.32 (0.82–6.38)	0.1
GATA4	High	2.70 (0.91–8.98)	0.07	2.83 (0.94–9.46)	0.06
Nuclear atypia	High	2.81 (1.01–7.38)	0.04*	2.91 (1.04–7.72)	0.04*
HER2 and GATA4	High + high	6.30 (1.85–24.59)	0.003**	8.75 (2.20–39.48)	0.002**

(C) Multivariate; Comparison of the molecular prognostic factors for DFS					
Factor	Level	HR (95% CI)	*P*-value	AHR (95% CI)	*P*-value
HER2	High	2.16 (0.79–5.82)	0.1	2.25 (0.77–6.32)	0.1
GATA4	High	3.02 (1.07–9.87)	0.04*	3.09 (1.06–10.14)	0.04*

(D) Univariate and multivariate risk factors for DSS					
	Univariate			Multivariate	
				
Factor	Level	HR (95% CI)	*P*-value	HR (95% CI)	*P*-value
Stage	II–III	6.27 (1.26–26.34)	0.03*	5.93 (1.12–27.86)	0.04*
GATA4	High	3.97 (1.14–18.21)	0.03*	2.82 (0.74–13.54)	0.1
Nuclear atypia	High	6.02 (1.72–23.59)	0.006**	6.56 (1.83–26.34)	0.004**

HR, hazard ratio; AHR, stage-adjusted hazard ratio; CI, confidence interval.

* *P < 0.05,

** *P* < 0.01.

In order to find independent prognostic factors as regards DFS, we carried out multivariate analysis using the significant prognostic factors shown in Table 3A. We found that nuclear atypia was an independent predictor of DFS, again also after adjusting for tumor stage (Table 3B). However, high-level coexpression of HER2 and GATA4 was an even stronger independent prognostic factor when analyzed with nuclear atypia (Table 3B), and also when analyzed only in stage I (HR 5.62, 95% CI 1.45–23.48, *P* = 0.01) and in stage Ia (HR 11.5, 95% CI 1.76–79.41, *P* = 0.01) tumors. However, in a multivariate comparison of the molecular prognostic factors, GATA4 was superior to HER2 in predicting DFS (Table 3C). The results were not affected by adjusting for different groups of tumor stage (Ia vs. Ib–III or I vs. II–III).

### Tumor stage, GATA4, and nuclear atypia are prognostic of DSS

**Figure 3:**
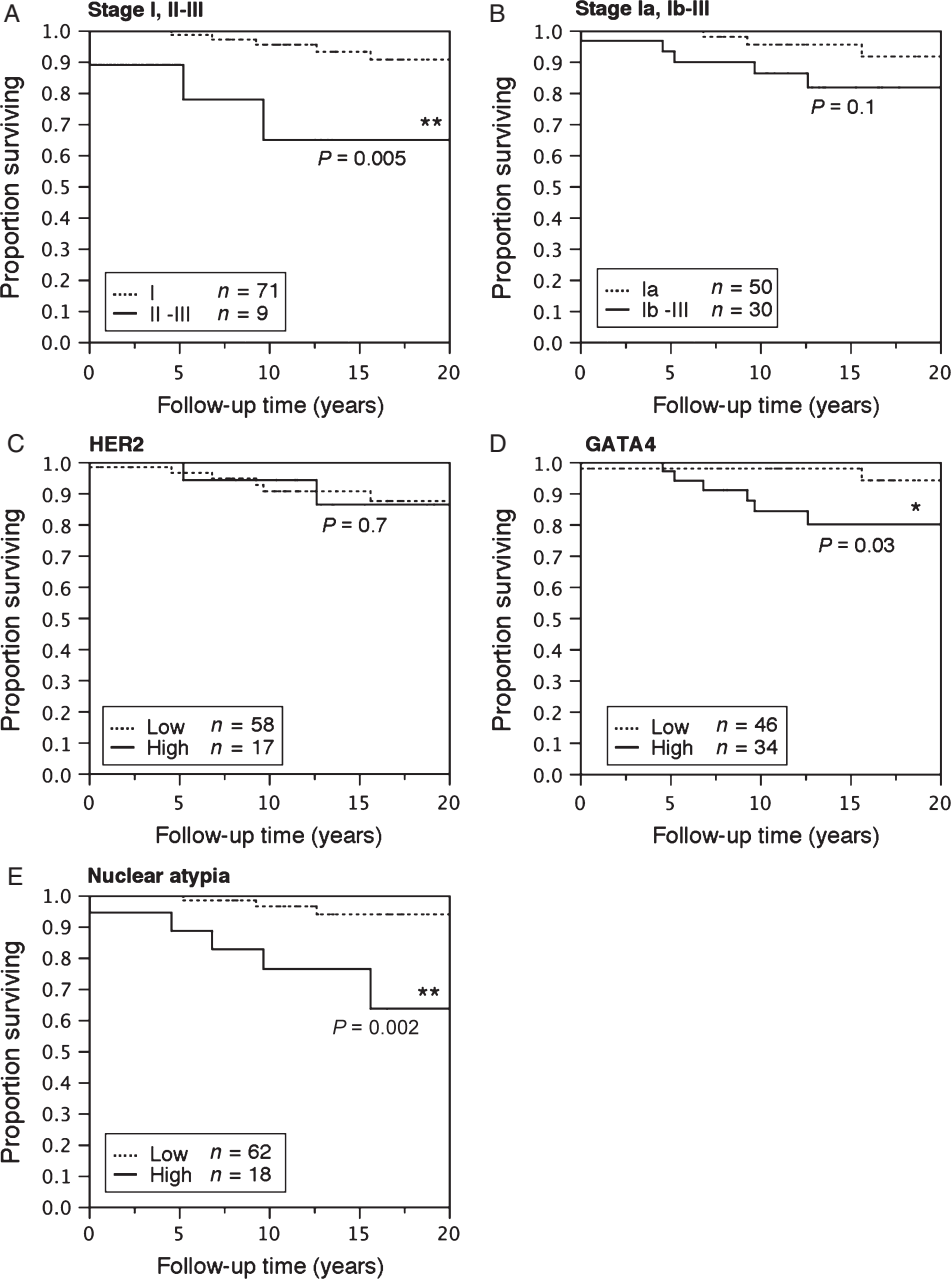
Kaplan–Meier plots of disease-specific survival (DSS) of GCT patients according to tumor stage, expression of HER2 and GATA4, and nuclear atypia. Proportion of patients surviving with either stage I or II–III tumors (A), and with tumors of stage Ia or Ib–II (B). Proportion of patients surviving with tumors expressing low or high levels of GATA4 (C) and HER2 (D) and with tumors having low- or high-level nuclear atypia (E). Log-rank test, differences between groups were considered significant when *P* < 0.05 (*P < 0.05, **P < 0.01).

In Kaplan–Meier analyses, patients with stage II–III tumors had shorter DSS (Fig. 3A, Table 3D) when compared with stage I patients. However, it must be noted that the number of stage II–III patients was relatively small (*n* = 9). DSS was similar in stage Ia patients when compared with those with stage Ib–III disease (Fig. 3B). Expression of HER2 was not associated with DSS (Fig. 3C). High-level GATA4 expression (Fig. 3D, Table 3D) and high-level nuclear atypia were prognostic as regards DSS (Fig. 3E, Table 3D). In multivariate analyses, stage II–III and high-level nuclear atypia both independently predicted poorer DSS (Table 3D).

## Discussion

An adult GCT is characterized by slow and indolent growth, but carries a risk of recurrence even relatively late after primary treatment. Tumor stage is the only prognostic factor in GCTs, however, in view of the fact that the majority of GCT patients are diagnosed with early-stage disease, a major challenge has been the identification of molecular prognostic markers to predict tumor recurrence. Overexpression of HER2 is an adverse prognostic factor in breast 14, gastric 34, pancreatic 35, lung 36, and ovarian carcinomas 13 and we previously reported that high-level expression of the transcription factor GATA4 is associated with more aggressive GCTs 27. Therefore, we now evaluated the prognostic significance of these factors in our large series of GCT patients with prolonged follow up. In addition, we assessed clinical prognostic factors as regards GCT recurrence and survival.

Because of the rarity and the long disease course of GCTs, prognostic factors have been difficult to establish 3,37. There are several advantages of this study when compared with previous studies on prognostic factors in GCTs. First, only histologically reconfirmed adult GCTs were included in the study; without reevaluation the rate of false original diagnoses of GCTs can be up to 50% 38. Second, an extended follow-up period is needed in order to detect all recurrent GCTs 10; we clinically examined over 30 patients in the follow-up study, and we were thus able to achieve a follow-up period of over 10 years in the majority (84%, *n* = 67) of the patients alive. This allowed us to reliably evaluate prognostic factors in most cases of possible recurrence. One disadvantage of this study is the small number of patients diagnosed with advanced tumors; there were no patients with stage IV GCTs, and only three patients with stage III tumors. However, this is a typical characteristic of GCT, and the numbers of early-stage tumors correspond to those reported in previous GCT studies 3,9.

In accordance with the results of previous studies 17–20, we found positive expression of HER2-4 in GCTs. HER2 was positive in the majority of GCTs, and also in its phosphorylated form. These findings contradict the results of some of the earlier studies where GCTs were to be negative of HER2 19,21–24. These mostly immunohistochemical studies involved the use of various methods and antibodies 19,21. In concordance with previous results 23, we found that *HER2* amplification is rare in GCTs. HER2 is a therapeutic target in breast and gastric cancers 16, and clinical studies concerned with targeting EGF receptors in epithelial ovarian cancer are ongoing (http://www.clinicaltrials.gov). GCTs respond to the targeting of EGF receptors HER3-4 with a cytotoxic ligand in vitro 17, and the results of this study imply that HER2 is also a potential target in the development of new treatment strategies for GCT patients, especially for patients with high-risk HER2-expressing tumors.

GATA4 is a crucial regulator of granulosa cell function 26,39 and putatively plays an important role in GCT pathogenesis by regulating GCT cell survival and apoptosis 28,29. The functional role of the pathognomonic FOXL2 mutation in GCTs is unknown, but it has been suggested to act as a tumor suppressor in granulosa cells by mediating apoptosis 40,41. Furthermore, recent molecular analyses suggest that the pathognomonic FOXL2 mutation causes imbalances in transforming growth factor (TGF)-*β* signaling, and more precisely, impaired interaction with SMAD transcription factors 42,43. GATA4 has been shown to interact with SMAD3 25, a member of the TGF-*β* signaling cascade, and recently also with FOXL2 44, thus linking GATA4 to the fundamental genetic changes in GCT pathogenesis. Moreover, GATA4 protein expression has been found to correlate positively with that of HER2 in breast cancer 45 and functional studies have shown that GATA4 directly binds *HER2* promoter and regulates its expression 46. In GCTs, we found that expression of GATA4 and HER2 strongly colocalized and together delineated an even more aggressive disease. Indeed, GATA4 may be one of the transcription factors mediating HER2 overexpression in GCTs. In addition, epigenetic changes may attribute to the biological roles of HER2 and GATA4 in GCTs. Increased promoter methylation of GATA4 has been shown in epithelial ovarian cancers 47. Furthermore, promoter methylation of HER2 may mediate chemo resistance in breast and lung cancers 48,49. Epigenetic modification of HER2–4 and GATA4 are unknown in GCTs. Promoter methylation of some genes (e.g., ER-α, BRCA1) has been shown to exist in GCTs 50, but their roles remain unknown. In this study, GATA4 was an independent prognostic factor in predicting GCT recurrence and also delineated survival of GCT patients. High-level expression of GATA4 in the primary tumor led to an average threefold increased recurrence risk, independently of tumor stage. In multivariate analyses, GATA4 was superior to HER2 in predicting DFS. These data suggest that GATA4 could be used as a single prognostic marker, including in early-stage GCTs.

On the basis of the present data, nuclear atypia seems to be the most potent factor in predicting DSS of GCT patients. However, one must keep in mind the fact that the number of GCT-related deaths was relatively small in this study. Furthermore, based on previous studies on GCTs, the prognostic role of nuclear atypia is controversial; in four studies it has been found to be associated with worse prognosis 51–54, whereas in two studies this has not been confirmed 55,56. Furthermore, nuclear atypia is an unspecific morphological characteristic associated with great inter- and intraobserver variation, underscoring the need for specific molecular markers for GCTs. In conclusion, we have shown that HER2 and GATA4 are new prognostic factors for GCT. It is to be hoped that the identification of solid prognostic markers, coupled with increasing understanding of GCT pathogenesis, will lead toward more targeted treatment and follow-up strategies for GCT patients.
